# Supplementary material for: A new basal ichthyosauromorph from the Lower Triassic (Olenekian) of Zhebao, Guangxi Autonomous Region, South China
Source: PeerJ. 2022 Apr 7;10:e13209. doi: 10.7717/peerj.13209 (PMC8995025; doi:10.7717/peerj.13209)
Supplement: Supplemental Information 1 [file peerj-10-13209-s001.docx]

**Table S1 Original data plotted in Figure 4, with sources. Measurements in parentheses were estimated based on photos in the cited sources.**

| **Group** | **Species** | **Specimen number** | **Numbers of centrum used for calculation** | **Centrum height (mm)** | **Centrum length (mm)** | **Reference** |
| --- | --- | --- | --- | --- | --- | --- |
| Basal Ichthyosauromorpha | *Nanchangosaurus suni* | GMC V 636 | 10, average | 7.6 | 3.6 | (Wang 1959) |
| Basal Ichthyosauromorpha | *Eohupehsuchus brevicollis* | WGSC V26003 | 3, average | (3.1) | (3.9) | (Chen et al. 2014a) |
| Basal Ichthyosauromorpha | *Hupehsuchus nanchangensis* | ZMNH M8127 | 3, average | (9) | (9.7) | (Carroll & Dong 1991) |
| Basal Ichthyosauromorpha | *Chaohusaurus brevifemoralis* | AGB7401 | 3, average | (6.7) | (8.3) | (Huang et al. 2019) |
| Basal Ichthyosauromorpha | *Chaohusaurus geishanensis* | IVPP V4001 | 3, average | (3.3) | (4.3) | (Young & Dong 1972) |
| Basal Ichthyosauromorpha | *Utatsusaurus hataii* | IGPS95941 | 10, average | 16.2 | 13.9 | (Shikama et al. 1978) |
| Basal Ichthyosauromorpha | *Grippia longirostris* | PMO 230.212 | 1 | (11.4) | (9.7) | (Roaldset 2017) |
| Basal Ichthyosauromorpha | *Gulosaurus helmi* | TMP 89.127.3 | 3, average | (8.6) | (6.1) | (Cuthbertson et al. 2013) |
| Basal Ichthyosauromorpha | *Omphalosaurus wolfi* | MBG 1500 | 1 | 75 | 58 | (Sander & Faber 2003) |
| Ichthyosauria | *Cymbospondylus buchseri* | PIMUZT 4351 | 4, average | (61.3) | (32.5) | (Sander 1989) |
| Ichthyosauria | *Cymbospondylus* sp. | PMO 229.734 | 1 | (44.2) | (23.7) | (Engelschiøn et al. 2018) |
| Ichthyosauria | *Cymbospondylus* sp. | PIMUZ A/III 496 | 1 | 61.5 | 30.8 | (Sander 1992) |
| Ichthyosauria | *Mixosaurus kuhnschnyderi* | YIGM SPCV-0810 | 1 | 3.6 | 2.5 | (Chen & Cheng 2009) |
| Ichthyosauria | *Qianichthyosaurus xingyiensis* | WS2011-46-R1 | 3, average | (17.5) | (11.7) | (Yang et al. 2013) |
| Ichthyosauria | *Shastasaurus neubigi* | BSP 1992 I 39 | 1 | 29 | 17 | (Sander 1997) |
| Ichthyosauria | *Ichthyosaurus communis* | NHMUK R15907 | 3, average | (11.3) | (6.7) | (Bennett et al. 2012) |
| Ichthyosauria | *Temnodontosaurus trigonodon* | SMNS 15950 | 3, average | (114.3) | (50.8) | (Pardo-Pérez et al. 2018) |
| Ichthyosauria | *Stenopterygius aaleniensis* | SMNS 90699 | 3, average | (44.2) | (20.1) | (Maxwell et al. 2012) |
| Ichthyosauria | *Gengasaurus nicosiai* | MSVG 39617 | 1 | 55.8 | 25.2 | (Paparella et al. 2016) |
| Ichthyosauria | *Muiscasaurus catheti* | FCG-CBP-16 | 3, average | (46.3) | (20.9) | (Paramo-Fonseca et al. 2021) |
| Sauropterygia | *Chinchenia sungi* | IVPP 3227 | 3, average | 9.8 | 14.1 | (Rieppel 1998a) |
| Sauropterygia | *Sanchiaosaurus dengi* | IVPP V2338 | 1 | 18.7 | 20.3 | (Rieppel 1998a) |
| Sauropterygia | *Corosaurus alcovensis* | UW 5485 | 3, average | (18.9) | (17.2) | (Rieppel 1998b) |
| Sauropterygia | *Augustasaurus hagdorni* | FMNH PR 1974 | 3, average | (36) | (40) | (Sander et al. 1997) |
| Sauropterygia | Eusauropterygia indet. | UCM-CLV34 | 1 | (30.8) | (22.5) | (Pérez-Valera et al. 2020) |
| Sauropterygia | *Yunguisaurus liae* | XNGMXY-2013-R1 | 3, average | (7.7) | (12.2) | (Wang et al. 2019) |

**Tables S2 Sources of morphological information for trunk elements of various taxa illustrated in Figure 6.**

| **Number** | **Group** | **Species** | **Specimen number** | **References** |
| --- | --- | --- | --- | --- |
| **A** | Ichthyosauromorpha | *Baisesaurus robustus* | CUGW VH107 |  |
| **B** | Ichthyosauromorpha | *Utatsusaurus hataii* | IGPS95941 | (Shikama et al. 1978) |
| **C** | Sauropterygia | *Lariosaurus sanxiaensis* | HFUT YZS-16-01 | (Li & Liu 2020) |
| **D** | Sauropterygia | *Dawazisaurus brevis* | NMNS000933-F034397 | (Cheng et al. 2016) |
| **E** | Thalattosauria | *Askeptosaurus italicus* | PIMUZ T 4832 | (Müller 2005) |
| **F** | Ichthyosauromorpha | *Baisesaurus robustus* | CUGW VH107 |  |
| **G** | Ichthyosauromorpha | *Chaohusaurus geishanensis* | P45-H85-23 | (Maisch 2001) |
| **H** | Ichthyosauromorpha | *Chaohusaurus chaoxianensis* | AGB6256 | (Huang et al. 2019) |
| **I** | Sauropterygia | *Kwangsisaurus orientalis* | IVPP V2338 | (Rieppel 1998a) |
| **J** | Sauropterygia | *Hanosaurus hupehensis* | IVPP V3231 | (Rieppel 1998c) |

**Tables S3 Sources of morphological information for forelimb elements of various taxa illustrated in Figure 7.**

| **Number** | **Group** | **Species** | **Specimen number** | **References** |
| --- | --- | --- | --- | --- |
| **A** | Ichthyosauromorpha | *Baisesaurus robustus* | CUGW VH107 |  |
| **B** | Ichthyosauromorpha | *Parahupehsuchus longus* | WGSC 26005 | (Chen et al. 2014b) |
| **C** | Ichthyosauromorpha | *Hupehsuchus nanchangensis* | ZMNH M8127 | (Carroll & Dong 1991) |
| **D** | Ichthyosauromorpha | *Eohupehsuchus brevicollis* | WGSC V26003 | (Chen et al. 2014a) |
| **E** | Ichthyosauromorpha | *Eretmorhipis carrolldongi* | WGSC V26020 | (Chen et al. 2015) |
| **F** | Ichthyosauromorpha | *Chaohusaurus brevifemoralis* | AGB7408 | (Huang et al. 2019) |
| **G** | Ichthyosauromorpha | *Chaohusaurus zhangjiawanensis* | WHGMR V26025 | (Chen et al. 2013) |
| **H** | Ichthyosauromorpha | *Grippia longirostris* | PMU R472 | (Zou et al. 2020) |
| **I** | Ichthyosauromorpha | *Utatsusaurus hataii* | IGPS95941 | (Mazin 1986) |
| **J** | Sauropterygia | *Majiashanosaurus discocoracoidis* | AGM-AGB5954 | (Jiang et al. 2014) |
| **K** | Sauropterygia | *Qianxisaurus chajiangensis* | NMNS-KIKO-F044630 | (Cheng et al. 2012) |
| **L** | Sauropterygia | *Dawazisaurus brevis* | NMNS000933-F034397 | (Cheng et al. 2016) |
| **M** | Thalattosauria | *Askeptosaurus italicus* | MSNM V456 | (Müller 2005) |

Bennett SP, Barrett PM, Collinson ME, Moore-Fay S, Davis PG, and Palmer CP. 2012. A new specimen of *Ichthyosaurus communis* from Dorset, UK, and its bearing on the stratigraphical range of the species. *Proceedings of the Geologists Association* 123:146-154.

Carroll RL, and Dong ZM. 1991. *Hupehsuchus*, an enigmatic aquatic reptile from the Triassic of China, and the problem of establishing relationships. *Philosophical Transactions of the Royal Society of London Series B: Biological Sciences* 331 (1260):131-153. DOI: 10.1098/rstb.1991.0004

Chen XH, and Cheng L. 2009. Discovery of *Mixosaurus* (Reptilia: ichthyosauria) in the Middle Triassic of Luoping, Yunnan (in Chinese with English abstract). *Acta Geologica Sinica* 83:1214-1220.

Chen XH, Sander PM, Cheng L, and Wang X. 2013. A new Triassic primitive ichthyosaur from Yuanan, South China. *Acta Geologica Sinica ‐ English Edition*:44-49. DOI: 10.1111/1755-6724.12078

Chen XH, Motani R, Cheng L, Jiang DY, and Rieppel O. 2014a. A small short-necked hupehsuchian from the Lower Triassic of Hubei Province, China. *PLOS ONE* 9:e115244. DOI: 10.1371/journal.pone.0115244

Chen XH, Motani R, Cheng L, Jiang DY, and Rieppel O. 2014b. A carapace-like bony ‘body tube’ in an Early Triassic marine reptile and the onset of marine tetrapod predation. *PLOS ONE* 9:e94396. DOI: 10.1371/journal.pone.0094396

Chen XH, Motani R, Cheng L, Jiang DY, and Rieppel O. 2015. A new specimen of Carroll's mystery hupehsuchian from the Lower Triassic of China. *PLOS ONE* 10:1-15. DOI: 10.1371/journal.pone.0126024

Cheng YN, Wu XC, Sato T, and Shan HY. 2012. A new eosauropterygian (Diapsida, Sauropterygia) from the Triassic of China. *Journal of Vertebrate Paleontology* 32:1335-1349.

Cheng YN, Wu XC, Sato T, and Shan HY. 2016. *Dawazisaurus brevis*, A new eosauropterygian From the Middle Triassic of Yunnan, China. *Acta Geologica Sinica ‐ English Edition* 90:401-424. DOI: 10.1111/1755-6724.12680

Cuthbertson RS, Russell AP, and Anderson JS. 2013. Cranial morphology and relationships of a new grippidian (Ichthyopterygia) from the Vega-Phroso Siltstone Member (Lower Triassic) of British Columbia, Canada. *Journal of Vertebrate Paleontology* 33:831-847. DOI: 10.1080/02724634.2013.755989

Engelschiøn VS, Delsett LL, Roberts AJ, and Hurum JH. 2018. Large-sized ichthyosaurs from the Lower Saurian niveau of the Vikinghøgda Formation (Early Triassic), Marmierfjellet, Spitsbergen. *Norwegian Journal of Geology* 98:239-265. DOI: 10.17850/njg98-2-05

Huang JD, Motani R, Jiang DY, Tintori A, and Zhang R. 2019. The new ichthyosauriform *Chaohusaurus brevifemoralis* (Reptilia, Ichthyosauromorpha) from Majiashan, Chaohu, Anhui Province, China. *PeerJ* 7:e7561. DOI: 10.7717/peerj.7561

Jiang DY, Motani R, Tintori A, Rieppel O, Chen GB, Huang JD, Zhang R, Sun ZY, and Ji C. 2014. The Early Triassic eosauropterygian *Majiashanosaurus discocoracoidis*, gen. et sp. nov. (Reptilia, Sauropterygia), from Chaohu, Anhui Province, People's Republic of China. *Journal of Vertebrate Paleontology* 34:1044-1052. DOI: 10.1080/02724634.2014.846264

Li Q, and Liu J. 2020. An Early Triassic sauropterygian and associated fauna from South China provide insights into Triassic ecosystem health. *Communications Biology* 3:1-11. DOI: 10.1038/s42003-020-0778-7

Maisch MW. 2001. Observations on Triassic ichthyosaurs. Part VII. New data on the osteology of *Chaohusaurus geishanensis* Young & Dong, 1972 from the Lower Triassic of Anhui (China). *Neues Jahrbuch für Geologie und Paläontologie - Abhandlungen* 219:305-327.

Maxwell EE, Fernández MS, and Schoch RR. 2012. First Diagnostic Marine Reptile Remains from the Aalenian (Middle Jurassic): A New Ichthyosaur from Southwestern Germany. *PLOS ONE* 7:e41692.

Mazin JM. 1986. A new interpretation of the fore-fin of *Utatsusaurus hataii* (reptilia, ichthyopterygia). *Palontologische Ztschrift* 60:313-318.

Müller J. 2005. The anatomy of *Askeptosaurus italicus* from the Middle Triassic of Monte San Giorgio and the interrelationships of thalattosaurs (Reptilia, Diapsida). *Revue Canadienne Des Sciences De La Terre* 42:1347-1367. DOI: 10.1139/e05-030

Paparella I, Maxwell EE, Cipriani A, Roncacè S, and Caldwell MW. 2016. The first ophthalmosaurid ichthyosaur from the Upper Jurassic of the Umbrian–Marchean Apennines (Marche, Central Italy). *Geological Magazine*:1-22.

Paramo-Fonseca ME, García-Guerrero J, Benavides-Cabra CD, Padilla-Bernal S, and Castaneda-Gomez AJ. 2021. A benchmark specimen of *Muiscasaurus catheti* from the Upper Aptian of Villa De Leiva, Colombia: new anatomical features and phylogenetic implications. *Cretaceous Research* 119:e104685.

Pardo-Pérez JM, Kear BP, Mallison H, Gómez M, and Maxwell EE. 2018. Pathological survey on *Temnodontosaurus* from the Early Jurassic of southern Germany. *PLOS ONE* 13:e0204951.

Pérez-Valera JA, Berrocal-Casero M, and Pérez-Valera F. 2020. First Triassic tetrapod (Eusauropterygia) in the Triassic of the Subbetic domain of the Betic Cordillera (Southeastern Spain). *PalZ* 94:343-352.

Rieppel O. 1998a. The sauropterygian genera *Chinchenia*, *Kwangsisaurus*, and *Sanchiaosaurus* from the Lower and Middle Triassic of China. *Journal of Vertebrate Paleontology* 19:321-337. DOI: 10.1080/02724634.1999.10011144

Rieppel O. 1998b. *Corosaurus alcovensis* case and the phylogenetic interrelationships of Triassic stem-group Sauropterygia. *Zoological Journal of the Linnean Society* 124:1-41. DOI: 10.1111/j.1096-3642.1998.tb00568.x

Rieppel O. 1998c. The systematic status of *Hanosaurus hupehensis* (Reptilia, Sauropterygia) from the Triassic of China. *Journal of Vertebrate Paleontology* 18:545-557. DOI: 10.1080/02724634.1998.10011082

Roaldset OF. 2017. Basal ichthyopterygian remains from the *Grippia* bonebed (Early Triassic) of Marmierfjellet, Spitsbergen M.Sc. Thesis. University of Oslo.

Sander PM. 1989. The large ichthyosaur *Cymbospondylus buchseri.* sp. nov., from the Middle Triassic of Monte San Giorgio (Switzerland), with a survey of the genus in Europe. *Journal of Vertebrate Paleontology* 9:163-173.

Sander PM. 1992. *Cymbospondylus* (Shastasauridae: Ichthyosauria) from the Middle Triassic of Spitsbergen: filling a paleobiogeographic gap. *Journal of Paleontology* 66:332-337.

Sander PM, Rieppel O, and Bucher H. 1997. A new pistosaurid (Reptilia: Sauropterygia) from the Middle Triassic of Nevada and its implications for the origin of the plesiosaurs. *Journal of Vertebrate Paleontology* 17:526-533.

Sander PM. 1997. The Paleobiogeography of Shastasaurus. *Ancient Marine Reptiles*, 17-43.

Sander PM, and Faber C. 2003. The Triassic marine reptile *Omphalosaurus*: Osteology, jaw anatomy, and evidence for ichthyosaurian affinities. *Journal of Vertebrate Paleontology* 23:799-816. DOI: 10.1671/6

Shikama T, Kamei T, and Murata M. 1978. Early Triassic Ichthyosaurus, *Utatsusaurus hataii* Gen. et Sp. Nov., from the Kitakami Massif, Northeast Japan. *Geological Institute, Tohoku University* 48:77-A42.

Wang K. 1959. Ueber eine neue fossile Reptiliform von Provinz Hupeh, China. *Acta Palaeontologica Sinica* 7:367-373.

Wang X, Lu H, Jiang DY, Zhou M, and Sun ZY. 2019. A new specimen of *Yunguisaurus* (Reptilia; Sauropterygia) from the Ladinian (Middle Triassic) Zhuganpo Member, Falang Formation, Guizhou, China and the restudy of *Dingxiaosaurus*. *Palaeoworld (2019)*. DOI: 10.1016/j.palwor.2019.05.006

Yang PF, Ji C, Jiang DY, Motani R, Tintori A, Sun YL, and Sun ZY. 2013. A new species of Middle Triassic *Qianichthyosaurus* (Reptilia: Ichthyosauria) from Xingyi, Guizhou Province (in Chinese with English abstract). *Journal of Peking University (natural science)* 49:1002-1008.

Young CC, and Dong ZM. 1972. *Triassic aquatic reptiles in China*: Beijing: Science Press (in Chinese wtih English abstract).

Zou YR, Zhao B, Chen G, Li JL, Cheng L, Yan CB, and Tan QM. 2020. New materials of ichthyosaur forelimbs from Early Triassic in Hubei Province and their correlation (in Chinese with English abstract). *Acta Geologica Sinica ‐ English Edition* 94:1017-1026. DOI: CNKI:SUN:DZXE.0.2020-04-002
